# Parent Perceptions of Trainees in Pediatric Care: Cross-Sectional Study

**DOI:** 10.2196/46631

**Published:** 2023-12-13

**Authors:** Haley Strouf Motley, Bradley Kerr, Daniel J Sklansky, Jens Eickhoff, Megan A Moreno, Jessica C Babal

**Affiliations:** 1 Department of Pediatrics University of Wisconsin School of Medicine and Public Health Madison, WI United States; 2 Department of Biostatistics and Medical Informatics University of Wisconsin School of Medicine and Public Health Madison, WI United States

**Keywords:** latent class analysis, medical student, resident, trainee, medical education, trust, comfort, parents, pediatrics, parent perception, pediatric care, clinical autonomy

## Abstract

**Background:**

Clinical experience and progressive autonomy are essential components of medical education and must be balanced with patient comfort. While previous studies have suggested that most patients accept trainee involvement in their care, few studies have focused specifically on the views of parents of pediatric patients or examined groups who may not report acceptance.

**Objective:**

This study aims to understand parental profiles of resident and medical student involvement in pediatric care and to use latent class analysis (LCA) methodology to identify classes of responses associated with parent demographic characteristics.

**Methods:**

We used data from a national cross-sectional web-based survey of 3000 parents. The survey used a 5-point Likert scale to assess 8 measures of parent perceptions of residents and medical students. We included participants who indicated prior experience with residents or medical students. We compared responses about resident involvement in pediatric care with responses about student involvement, used LCA to identify latent classes of parent responses, and compared demographic features between the latent classes.

**Results:**

Of the 3000 parents who completed the survey, 1543 met the inclusion criteria for our study. Participants reported higher mean scores for residents than for medical students for perceived quality of care, comfort with autonomously performing an examination, and comfort with autonomously giving medical advice. LCA identified 3 latent classes of parent responses: Trainee-Hesitant, Trainee-Neutral, and Trainee-Supportive. Compared with the Trainee-Supportive and Trainee-Neutral classes, the Trainee-Hesitant class had significantly more members reporting age <30 years, household income < US $50,000, no college degree, and lesser desire to receive future care at a teaching hospital (all *P*<.05).

**Conclusions:**

Parents may prefer greater clinical autonomy for residents than medical students. Importantly, views associated with the Trainee-Hesitant class may be held disproportionately by members of historically and currently socially marginalized demographic groups. Future studies should investigate underlying reasons for trainee hesitancy in these groups, including the possibility of mistrust in medicine.

## Introduction

Trusting, collaborative relationships between patients and health care providers are associated with improved health outcomes, trust in medical systems, and patient empowerment [1,2]. One factor that may affect patient trust in medical systems is trainee involvement in patient care. Positive interactions with trainees can improve patient perceptions of trainees and trust in medical systems [3]. Previous studies have demonstrated adult patients’ interest in participating in medical education [3-7].

Notably, however, adult patients generally report a preference for working with trainees who are more advanced in training [4-7]. Additionally, patients report a preference for lower extent of trainee autonomy, preferring for trainees to have history-taking autonomy over medical decision-making autonomy, for example [4-7]. Understanding patient perceptions of trainees in pediatric settings may be more complex than in adult settings, as pediatric care separates the patient from the decision-making individual [8]. Parents may have concerns about their child’s safety in the context of trainee competence [9,10].

Clinical experience and progressive autonomy are crucial to medical training [11,12]. Clinical experiences involving diverse populations, resource levels, and medical complexity contribute to trainee skills, confidence, and career goals [3,12-14]. Given the importance of promoting progressively autonomous trainee clinical experiences across diverse backgrounds, it is important to understand how trainee involvement affects parent comfort and perceptions in pediatric care. However, parental views on trainee involvement remain underexplored, especially regarding how those views may vary across parent demographic backgrounds.

This study aimed to understand parental perceptions of resident and medical student involvement in pediatric care, using latent class analysis (LCA) methodology to identify response patterns associated with parent demographic characteristics.

## Methods

This study used data from a larger national cross-sectional survey conducted via the web-based Qualtrics platform in October 2018.

### Participants and Recruitment

We aimed to recruit a national sample of participants who identified as parents of children younger than 18 years. To achieve this, we used a web-based survey panel. Studies have shown survey panels to offer faster data collection, broader geographic reach, more efficient analysis, and easier replication than traditional survey approaches [15]. We selected the Qualtrics survey platform for its ability to achieve demographic attributes within 10% of their corresponding US population values [16]. When panelists join Qualtrics, they complete demographic assessments so that survey invitations can be targeted to eligible survey populations.

A Qualtrics survey manager sent email invitations to English-speaking adult panel participants who reported having at least 1 child younger than 18 years. Eligible participants were then asked if their child had a chronic or ongoing health condition. To ensure a diverse array of prior experience with trainees and adequate representation of parents of children with medical complexity, we further asked Qualtrics to oversample for parents of children with a chronic or ongoing condition. These data were collected as part of a larger survey project, and sample size estimates were based on analyses for other variables at 3000 participants. Survey responses were reviewed, and atypical responses were removed and replaced in the data set by the Qualtrics manager. Participants had the option to opt out of individual survey items, but entirely blank sections would lead to replacement in the data set by the Qualtrics manager. The survey closed when a total sample size of 3000 parents was achieved, with at least 750 (25%) parents reporting a chronic or ongoing condition. For this analysis, we included participants who indicated prior experience with residents or medical students and responded to all survey items.

### Survey Procedures

Adaptive questioning was used to reduce the number and complexity of questions. Participants received approximately 5 pages of survey items with an average of 8 items per page, depending on skip logic for individual participants. Participants were able to review and change their answers before final submission of the survey. Survey items were not randomized.

### Measurements

Perceptions of trainee involvement were assessed using 2 statement sets adapted from a previously used multispecialty patient perception survey [5]. Parents and pediatricians pilot-tested and reviewed the adapted items before final revision. Statement sets assessed perceived quality of care with trainee involvement (Quality); acceptability of extending the visit to accommodate trainee participation (Visit Length); comfort with trainee autonomy (Autonomy) in performing a history of present illness (HPI), physical examination (Exam), and offering medical advice (Advice); perceived importance of their interaction to trainee education (Importance); enjoyment working with the trainee (Enjoyment); and desire to receive future care at a teaching hospital (Teaching Hospital). The survey began with the following definitions of medical students and resident physicians: “Medical students have graduated from college and are studying to become a doctor. Resident physicians are doctors who have graduated from medical school and are training to become specialized in fields such as pediatrics and family medicine.” Parents then received 1 set of statements about perceptions of resident care and an identical set about perceptions of medical student care (Table 1). Participants were asked to indicate their agreement with each statement using a 5-point Likert scale from “strongly disagree” to “strongly agree.” Participants were also asked to indicate their prior experience with residents and medical students and the clinical settings in which the interactions occurred (clinic, hospital, emergency department, and others).

**Table 1:** Qualtrics survey items assessing parent perceptions of medical trainee^a^ participation in their children’s care^b^.

Category and item shorthand			Survey item
**Perceived quality**			
	Quality	In general, trainee involvement in my child’s care improves the quality of my child’s care.	
**Acceptability of extending the visit**			
	Visit Length	In general, I would be comfortable with the medical visit lasting slightly longer (approximately 20 minutes longer) so that a trainee could be part of my child’s care.	
**Comfort with trainee autonomy**			
	HPI^c^	In general, I am comfortable with trainees asking questions about my child’s health history without a supervising physician present.	
	Exam	In general, I am comfortable with trainees examining my child without a supervising physician present.	
	Advice	In general, I am comfortable with trainees offering medical advice about my child without a supervising physician present.	
**Importance**			
	Importance	In general, allowing trainees to help manage my child’s care is important for trainee education.	
**Enjoyment**			
	Enjoyment	Overall, I have enjoyed my experience with trainee involvement in my child’s care.	
**Teaching hospital preference**			
	Teaching Hospital	If given the choice between my child receiving care at a teaching hospital (with medical students or resident physicians) or a nonteaching hospital (without medical students or resident physicians), I would prefer a teaching hospital.	

^a^“Trainee” indicates a medical student or resident physician. Each type of trainee was referenced specifically in its own set of questions and defined for participants as follows: “Medical students have graduated from college and are studying to become a doctor. Resident physicians are doctors who have graduated from medical school and are training to become specialized in fields such as pediatrics and family medicine.”

^b^The national web-based survey was distributed to English-speaking adults with at least 1 child younger than 18 years in October 2018.

^c^HPI: history of present illness.

Demographic variables included age, sex, race, ethnicity, highest level of education, household income, oldest child’s age, chronic or ongoing conditions, US geographic region, community size, and primary health care provider specialization (eg, pediatrics and family medicine).

### Analysis

Analyses were conducted using SAS software (version 9.4; SAS Institute). All reported *P* values were 2-sided. Statistical significance was defined using *P*<.05.

### Comparisons

We performed descriptive analyses, comparing parent responses about resident involvement in pediatric care with parent responses about student involvement using 2-sample 2-tailed *t* tests or nonparametric Wilcoxon rank sum tests.

### LCA

LCA is a statistical method of identifying unmeasured “class membership” among subjects using categorical observed variables [17]. It produces mutually exclusive latent classes of individuals based on their responses to observed variables [18]. LCA class membership is based on overall patterns of individual participant responses and verified with mathematical evaluation against a proposed model, distinguishing it from other methods such as factor analysis or clustering techniques that rely on specific variable cutoffs. LCA identifies classes of similar subjects while allowing for heterogeneity in individual variables, enabling participants to be grouped by response distribution “shape” rather than exclusively by response “magnitude” [18,19]. It is therefore often considered a more “person-centered” approach than more traditional “variable-centered” techniques [19]. The LCA was conducted under the model assumption of conditional independence, which assumes that variables within latent classes are independent of one another. This was validated by evaluating the model fit in our analyses.

We performed LCA using the following items for residents and students separately as input parameters: Quality, Visit Length, Autonomy (average of HPI, Exam, and Advice), Importance, and Enjoyment. Parameter estimation was performed using the maximum likelihood method. The number of classes was determined by conducting an iterative analysis approach, starting with a model with 2 (*k*) classes. The Vong-Lo-Mendell-Rubin (VLMR) likelihood ratio test was used to compare the model with *k* classes to a model with *k* – 1 classes. A significant *P* value (*P*<.05) indicates that the model with *k* classes provides a better model fit than the corresponding model with only *k* – 1 classes. Furthermore, model fit was also evaluated by comparing model fit statistics, including the Akaike information criteria (AIC) and Bayesian information criteria (BIC), between the model with *k* classes and the corresponding model with *k* – 1 classes. If the AIC and BIC of the model with *k* classes were smaller than those of the model with *k* – 1 classes, then the model with *k* classes indicated a better model fit. This analysis was repeated until there was an indication of improvement in model fit. Classification diagnostics were examined by evaluating the entropy and the diagonal elements of the posterior probability matrix. An entropy value and diagonal elements of the posterior probability matrix greater than 0.8 are generally considered acceptable.

After identifying latent classes, we compared class member demographic features between classes using *χ*^2^ analysis or the Wilcoxon rank sum test. The LCA was conducted using the Mplus software (version 8; Muthen & Muthen).

### Ethical Considerations

The University of Wisconsin-Madison Education and Social/Behavioral Science Institutional Review Board approved the study (2018-1051). Eligible participants were informed of the survey length, investigator, and study purposes before providing consent and voluntarily beginning the survey. No identifiable personal information was collected. We used a standard Qualtrics compensation feature in which participants receive “Qualtrics Points” as incentives for survey completion. These points can be accumulated across multiple surveys and can be applied toward purchases such as gift cards and airline miles.

## Results

### Participant Characteristics

Of the 1534 participants who met the inclusion criteria for this study, 1120 (73%) participants identified as non-Hispanic White, 1292 (84.8%) participants identified as female, and 652 (42.5%) participants reported having a child with a chronic condition (Table 2).

**Table 2:** Demographic characteristics of overall Qualtrics survey participants and participants included in LCA^a^ analysis^b^.

Characteristic			Overall survey (N=3000), n (%)		LCA participants (n=1534), n (%)
**Age (y)**					
	<20	14 (0.6)		5 (0.4)	
	20-30	550 (21.7)		270 (20.8)	
	30-40	1059 (41.7)		532 (40.9)	
	>40	915 (36.1)		493 (37.9)	
**Sex**					
	Female	2621 (87.9)		1292 (84.8)	
	Male	360 (12.2)		231 (15.2)	
**Ethnicity**					
	Hispanic	338 (11.3)		183 (12)	
	Non-Hispanic	2645 (88.7)		1339 (88)	
**Race**					
	Asian	90 (3)		37 (2.4)	
	Black	266 (8.9)		141 (9.3)	
	White	2466 (82.5)		1254 (82.2)	
	Another racial identity, not listed	167 (5.6)		93 (6.1)	
**Highest level of education**					
	College degree	900 (30.1)		492 (32.2)	
	No college degree	2093 (69.9)		1036 (67.8)	
**Household income, (US $)**					
	<50,000	1410 (47.2)		711 (46.5)	
	50,000-100,000	856 (28.6)		421 (27.5)	
	>100,000	724 (24.2)		398 (26)	
**Age of oldest child (y)**					
	<12	1796 (59.9)		875 (57.1)	
	≥12	1204 (40.1)		658 (42.9)	
**Chronic condition**					
	Yes	750 (25)		651 (42.5)	
	No	2250 (75)		883 (57.5)	
**Region of residence**					
	Midwest	756 (25.6)		409 (27.2)	
	Northeast	518 (17.6)		274 (18.2)	
	South	1199 (40.6)		598 (39.8)	
	West	477 (16.2)		223 (14.8)	
**Community size**					
	Rural	1050 (35.3)		525 (34.6)	
	Suburban	1289 (43.4)		639 (42.1)	
	Urban	634 (21.3)		354 (23.3)	
**Primary health care provider specialty**					
	Pediatrics	2100 (70.1)		1041 (68)	
	Nonpediatrics	896 (29.9)		489 (32)	
**Prior experience with trainees caring for their child**					
	None	1046 (34.9)		0 (0)	
	Medical student only	382 (12.7)		194 (15.1)	
	Resident physician only	568 (18.9)		509 (39.5)	
	Both	585 (19.5)		585 (45.4)	
**The setting of interaction with trainee**					
	Clinic	1255 (41.8)		850 (55.4)	
	Hospital	860 (28.7)		585 (38.1)	
	Emergency department	471 (15.7)		311 (20.3)	
	Other	29 (1)		25 (1.6)	

^a^LCA: latent class analysis.

^b^All participants were English-speaking adults with at least 1 child younger than 18 years and completed the national web-based survey in October 2018.

### Descriptive Analysis

**Figure 1:**
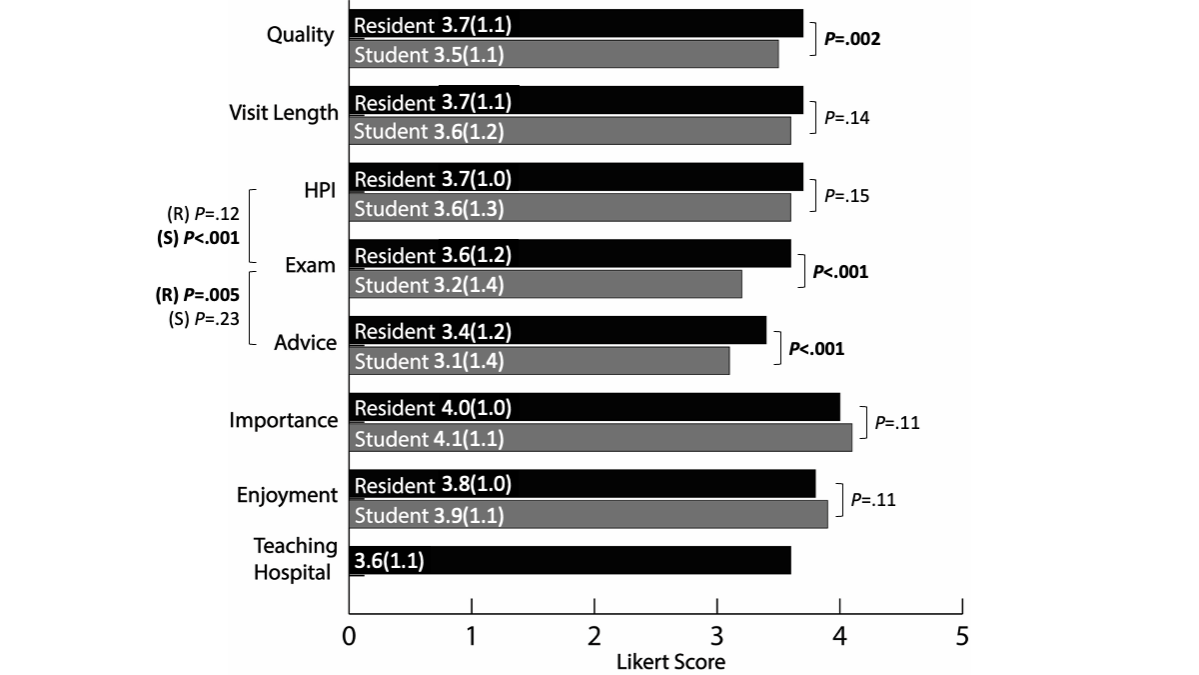
Aggregate analysis of parent perceptions of resident physicians and medical student participation in their child’s care. All participants were English-speaking adults with at least 1 child younger than 18 years and completed the national web-based survey in October 2018. The numbers provided in bars represent the mean (SD) Likert score. HPI: history of present illness.

Participants reported higher mean scores for residents than for medical students regarding perceived quality of care (resident: mean 3.7, SD 1.1; student: mean 3.5, SD 1.1; *P*=.002), comfort with autonomously performing an examination (resident: mean 3.6, SD 1.2; student: mean 3.2, SD 1.4; *P*<.001), and comfort with autonomously giving medical advice (resident: mean 3.4, SD 1.2; student: mean 3.1, SD 1.4; *P*<.001). The mean score for the desire to receive care at a teaching hospital was 3.6 (SD 1.1; Figure 1).

For each trainee type (resident and student), participants reported higher mean scores for comfort with HPI Autonomy than for Exam Autonomy (resident: *P*=.12, student: *P*<.001). Participants reported higher mean scores for Exam Autonomy than for Advice Autonomy (resident: *P*=.005, student: *P*=.23).

### LCA

**Figure 2:**
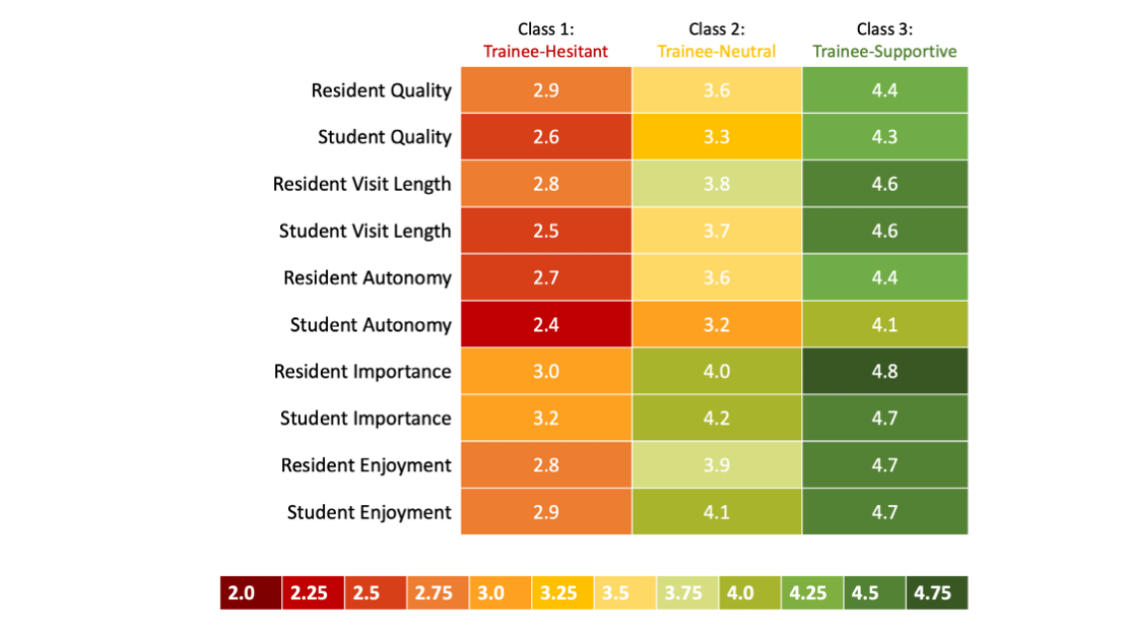
Latent class analysis of parent responses to survey items assessing perceptions of trainees participating in their child’s care. All participants were English-speaking adults with at least 1 child younger than 18 years and completed the national web-based survey in October 2018. The numbers shown represent each class’s mean Likert score survey items in that category.

Based on the results of the VLMR likelihood ratio test in combination with model fit evaluation, a 3-class model structure was determined as the final LCA model. The *P* values of the VLMR likelihood ratio test for comparing an LCA model with *k* classes to a model with *k* – 1 classes for *k*=2, 3, and 4 were *P*<.001, *P*=.15, and *P*=.76, respectively, suggesting a 2- or 3-class structure. The AIC and BIC model fit values for the model with 3 classes were significantly smaller than those for the model with 2 classes, with AIC values of 21,952 versus 23,014 and BIC values of 22,506 versus 23,302*.* The entropy value is 0.81, while the diagonal elements of the posterior probability matrix for the 3 classes are 0.91, 0.91, and 0.92 respectively, indicating desirable levels of classification properties. The 3 distinct classes had similar response patterns, with Student Autonomy consistently receiving the lowest mean Likert score and Student or Resident Important receiving the highest mean Likert score. The classes were primarily distinguished by overall magnitude in Likert scores and are therefore denoted as “Trainee-Hesitant” (n=494), “Trainee-Neutral” (n=668), and “Trainee-Supportive” (n=370) classes. The Trainee-Hesitant class reported mean question set scores in perceptions of trainee involvement ranging from 2.4 to 3.2. The Trainee-Neutral class reported scores ranging from 3.2 to 4.2, and the Trainee-Supportive class reported scores ranging from 4.1 to 4.8 (Figure 2).

Compared with the Trainee-Supportive and Trainee-Neutral classes, the Trainee-Hesitant class had significantly more members reporting age <30 years (Trainee-Supportive: *P*=.004; Trainee-Neutral: *P*=.01), household income <US $50,000 (Trainee-Supportive: *P*<.001; Trainee-Neutral: *P*<.001), no college degree (Trainee-Supportive: *P*<.001; Trainee-Neutral: *P*<.001), and lesser desire to receive future care at a teaching hospital (Trainee-Supportive: *P*<.001; Trainee-Neutral: *P*<.001).

Compared with the Trainee-Supportive class, the Trainee-Hesitant class had significantly more members reporting race other than White (*P*=.003), Hispanic or Latinx ethnicity (*P*=.003), and multiple children (*P*=.04).

Compared with the Trainee-Supportive class, the Trainee-Neutral class members reported significantly lower household income (*P*<.001), lower education (*P*<.001), more children (*P*=.04), more pediatric primary care providers (*P*=.03), and lower teaching hospital preference (*P*<.001; Table 3).

**Table 3:** Comparison of parent demographic characteristics between latent classes^a^.

Characteristic		Class^b^				*P* value		
		TH^c^ (n=494), n (%)	TN^d^ (n=668), n (%)	TS^e^ (n=370), n (%)	TH vs TN		TN vs TS	TH vs TS
**Race**								
	White	380 (77.4)	548 (82.5)	326 (88.1)	.03		.02	<.001
	Non-White	111 (22.6)	116 (17.5)	44 (11.9)	.03		.02	<.001
**Ethnicity**								
	Hispanic	76 (15.5)	73 (11.0)	34 (9.2)	.02		.36	.006
	Non-Hispanic	414 (84.5)	590 (89.0)	335 (90.8)	.02		.36	.006
**Household income (US $)**								
	<50,000	265 (53.5)	300 (45.0)	146 (39.7)	.004		.10	<.001
	50,000-100,000	120 (24.2)	212 (31.8)	89 (24.2)	.005		.01	.98
	>100,000	110 (22.2)	155 (23.2)	133 (36.1)	.68		<.001	<.001
**Highest level of education**								
	No college degree	386 (78.3)	429 (64.3)	221 (60.1)	<.001		.17	<.001
	College degree	107 (21.7)	238 (35.7)	147 (39.9)	<.001		.17	<.001
**Parent age (y)**								
	<30	105 (25.9)	105 (18.5)	65 (20.6)	.005		.42	.10
	30-40	176 (43.5)	242 (42.5)	114 (36.2)	.76		.07	.049
	>40	124 (30.6)	223 (39.1)	136 (43.2)	.01		.24	<.001
**Sexual orientation**								
	Heterosexual	440 (89.2)	594 (89.3)	320 (86.7)	.97		.21	.26
	Not heterosexual	53 (10.8)	71 (10.7)	49 (13.3)	.97		.21	.26
**Region of residence**								
	Midwest	118 (24.2)	193 (29.4)	98 (27.2)	.05		.47	.32
	Northeast	91 (18.7)	100 (15.2)	83 (23.1)	.12		.002	.12
	South	186 (38.2)	273 (41.6)	139 (38.6)	.25		.36	.90
	West	92 (18.9)	91 (13.9)	40 (11.1)	.02		.21	.002
**Number of children**								
	1	145 (29.3)	199 (29.8)	133 (35.9)	.85		.04	.04
	>1	350 (70.7)	469 (70.2)	237 (64.1)	.85		.04	.04
**Chronic condition**								
	No	294 (59.4)	388 (58.1)	200 (54.1)	.65		.21	.12
	Yes	201 (40.6)	280 (41.9)	170 (45.9)	.65		.21	.12
**Teaching hospital**								
	Agree	52 (14.4)	289 (57.2)	217 (77.0)	<.001		<.001	<.001
	Disagree	308 (85.6)	216 (42.8)	65 (23.0)	<.001		<.001	<.001

^a^All participants were English-speaking adults with at least 1 child younger than 18 years and completed the national web-based survey assessing their views of medical trainees in their child’s care in October 2018.

^b^Numbers are expressed as n (%), calculated as the percentage of participants who completed the specific demographic question.

^c^TH: Trainee-Hesitant class.

^d^TN: Trainee-Neutral class.

^e^TS: Trainee-Supportive class.

## Discussion

### Principal Findings

This national cross-sectional study supports that parents may feel more comfortable with resident autonomy than with student autonomy and may believe that residents enhance the quality of pediatric care more than students do. However, the findings also suggest that many parents recognize their family’s importance in contributing to trainee education and enjoy participating in trainee education. Finally, the LCA revealed 3 distinct classes of parent responses, including a class with negative perceptions that was associated with many historically and currently marginalized social demographic characteristics.

The findings that parents had more comfort with advanced trainees and less comfort with autonomy in complex patient care tasks (eg, giving medical advice vs performing a history) are consistent with nonpediatric studies [4-7]. Some pediatric studies have found that parents are willing to participate in medical education provided it does not compromise their child’s care [10,20]. Concerns about emotional distress or pain may lead parents to refuse any trainee participation [9]. These perceptions may reflect patient concerns about safety or trainee competence. We did not find evidence that trainee involvement is viewed differently by parents of pediatric patients than by patients treated in other medical situations.

While most participants reported neutral attitudes toward trainee involvement, majority-positive and majority-negative views were expressed by the Trainee-Supportive and Trainee-Hesitant response classes, respectively. Parents in the Trainee-Hesitant class were more likely to identify as a race other than White, identify as Hispanic or Latinx ethnicity, report lower household income, and lack a college degree, suggesting that they were more likely to be members of socially marginalized groups such as racial and ethnic minority groups or low–socioeconomic status households. They were also the least likely to indicate a desire to receive future care at a teaching hospital. These findings are consistent with other studies showing racial and ethnic disparities in health care use [21].

Our findings may reflect historically strained relationships between socially marginalized groups and medical institutions. In some studies, members of racial and ethnic minority groups report less trust in physicians, health insurance, and hospitals than their White peers, for example [22-24]. Similarly, studies have shown that patients identifying as low–socioeconomic status or racial and ethnic minority individuals report higher perceived bias in health care interactions than patients who do not identify as members of those groups [23,25]. Moreover, patient trust and experiences with health care providers are often associated with health care use and health outcomes [1,2,24].

The mistrust of trainees may mirror mistrust in the medical institution in general, suggested by lower teaching hospital preferences among parents in the Trainee-Hesitant class. Thus, solutions to improve perceptions of trainee involvement among socially marginalized groups may be similar to those proposed to improve trust in medical institutions. These approaches might include expanding medical student and resident education, increasing institutional diversity, promoting and monitoring equity in care delivery, addressing gaps in health literacy, and engaging in community partnerships. Diversity, equity, and inclusion across all roles in health care teams promote high-quality, cost-effective, and patient-centered care [26]. In the long term, community-specific concordance between patients and health care providers may help achieve equitable health outcomes [27,28]. Institutional efforts in the recruitment and retention of diverse faculty and students can help achieve this goal [29,30]. In the meantime, while significant disparities exist among trainees and faculty alike [31,32], it is especially important to adopt educational strategies that cultivate mutuality, collaboration, and trust between trainees and patients [33,34]. In addition to medical staff and trainee education, there may also be roles for health literacy initiatives such as patient education materials defining members of the health care team [7] or badge systems that identify trainees and other team members in clinical settings [35]. Critically, any interventions adopted must be evidence-based, developed in partnership with the communities they serve, and continuously evaluated and improved [33].

Our study had limitations to consider. Using English-speaking eligibility criteria increased the feasibility of our study but likely limited non–English-speaking parent participation. Non-English speakers also experience health disparities and report higher rates of medical mistrust than their English-speaking peers [24,36,37]. This may limit the generalizability of our study. Translating future surveys into other languages or partnering with specific communities to directly assess the perceptions of non–English-speaking parents may help address this gap. While our study did achieve a geographically diverse sample, the demographic distribution of respondents was skewed toward female and non-Hispanic White participants relative to the US population. However, the high proportion of women may be reflective of the gendered distribution of childcare responsibilities, including attendance at children’s medical appointments [38]. Similarly, the high proportion of non-Hispanic White participants may reflect racial and ethnic disparities in access to and use of health care [21,39]. Regardless of etiology, it is possible that this demographic skew overlooks gender-specific or cultural views of health and wellness, including medical trainees. Community- or institution-specific studies may achieve a more representative sample and mitigate the effects of barriers to health care on study participation. Additionally, the survey was conducted using a web-based panel that is subject to sampling bias with voluntary participants who may share distinct characteristics of internet respondents. Moreover, this method of sampling made it impossible to calculate a response rate. In addition to explicitly recruiting diverse and representative sample populations, future studies should examine facilitators of negative perceptions of trainees and explore how these parents’ views alter health behaviors such as care seeking and medical adherence, paying particular attention to social factors that may influence parental comfort with care provision.

### Conclusions

Understanding gaps in parent comfort with trainee involvement in pediatric care will support teaching institutions in their aims to build trust and collaboration between patients and trainees and provide equitable patient care. LCA revealed that majority-negative views of trainee involvement in pediatric care may be disproportionately held by members of historically and currently socially marginalized demographic groups. Further investigation is needed to better understand these views, identify their upstream causes and downstream effects, and develop potential interventions to address them.

## Abbreviations

AIC: Akaike information criteria
BIC: Bayesian information criteria
HPI: history of present illness
LCA: latent class analysis
VLMR: Vong-Lo-Mendell-Rubin

## Data Availability

The data set analyzed during this study is available from the corresponding author on reasonable request.
